# Supplementary material for: Longitudinal automated brain volumetry versus expert visual assessment of atrophy progression on MRI: an exploratory study
Source: Sci Rep. 2025 Apr 29;15:14968. doi: 10.1038/s41598-025-98360-x (PMC12041260; doi:10.1038/s41598-025-98360-x)
Supplement: Supplementary file 1 — Supplementary Information. [file 41598_2025_98360_MOESM1_ESM.pdf]

Supplementary materials

Supplementary Table S1. Device settings for 3D T1 MPRAGE on different MRI-scanners.

MRI scanner*	Inversion time (ms)	Flip angle (°)	Repetition time (ms)	Pixel bandwidth	Number of imaging per scanner
3T Biograph mMR PET-MR system	1040	9	1830	180	12
3T MAGNETOM Prisma	900	8	1800	160	14
3T MAGNETOM Trio	900	9	1900	199	14

*Devices are from Siemens Healthineers/Healthcare GmbH, Erlangen Germany. Abbreviations: T, Tesla, magnetic field strength.

Supplementary Table S2: Summary table of anatomical regions provided by the AIRC-tool that were grouped for comparison with expert visual assessment-score.

frontal lobe	parietal lobe	left temporal lobe	right temporal lobe	left hippo-campus	right hippo-campus	ventricles
frontal GM right	parietal GM right	temporal GM left	temporal GM right t	hippo-campus left	hippo-campus right	lateral ventricle left
frontal GM left	parietal GM left	temporal WM left	temporal WM right			lateral ventricle right
frontal WM right	parietal WM right					3rd ventricle
frontal WM left	parietal WM left					

Abbreviations: GM: gray matter, WM: white matter

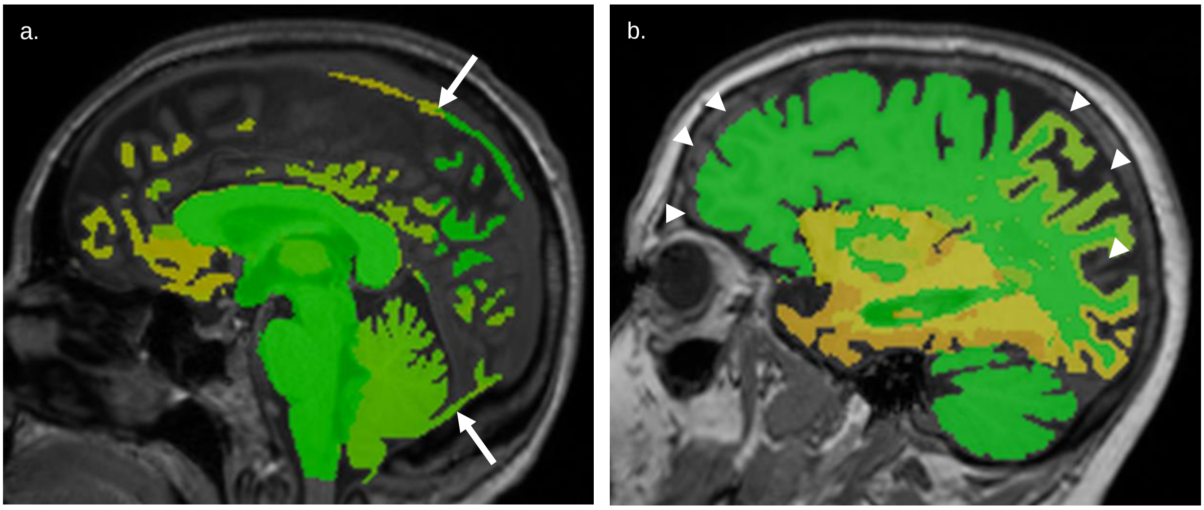

Supplementary Figure S1. Examples of segmentation irregularities. Fused image of 3D T1 MPRAGE and color-coded deviation map (z-scores) provided by the AIRC tool. a. Arrows pointing to "plus-variant", where the AIRC-tool included parts of the falx cerebri and venous sinuses. b. Arrowheads pointing to "minus-variant", where the tool missed parts of the frontal and parietooccipital lobes.
